# Microwave-Assisted Extraction for Microalgae: From Biofuels to Biorefinery

**DOI:** 10.3390/biology7010018

**Published:** 2018-02-15

**Authors:** Rahul Vijay Kapoore, Thomas O. Butler, Jagroop Pandhal, Seetharaman Vaidyanathan

**Affiliations:** Department of Chemical and Biological Engineering, The University of Sheffield, Sheffield S1 3JD, UK; J.pandhal@sheffield.ac.uk (J.P.); S.vaidyanathan@sheffield.ac.uk (S.V.)

**Keywords:** biorefinery, microalgae, microwave-assisted extraction (MAE), lipid extraction, direct transesterification (DT), biofuels

## Abstract

The commercial reality of bioactive compounds and oil production from microalgal species is constrained by the high cost of production. Downstream processing, which includes harvesting and extraction, can account for 70–80% of the total cost of production. Consequently, from an economic perspective extraction technologies need to be improved. Microalgal cells are difficult to disrupt due to polymers within their cell wall such as algaenan and sporopollenin. Consequently, solvents and disruption devices are required to obtain products of interest from within the cells. Conventional techniques used for cell disruption and extraction are expensive and are often hindered by low efficiencies. Microwave-assisted extraction offers a possibility for extraction of biochemical components including lipids, pigments, carbohydrates, vitamins and proteins, individually and as part of a biorefinery. Microwave technology has advanced since its use in the 1970s. It can cut down working times and result in higher yields and purity of products. In this review, the ability and challenges in using microwave technology are discussed for the extraction of bioactive products individually and as part of a biorefinery approach.

## 1. Introduction

To date, the total number of algal taxa which exist is unknown, but Guiry [1] reported that there could be at least 72,500 species with around 30,000 species of microalgae [2]. Since the 1970s the ambition has been to utilise microalgae for biofuels due to concerns regarding oil security and a requirement for reduction in the greenhouse gas emissions, and this has been extensively reviewed [3,4,5]. Similar to land-based plants, microalgae do offer the potential for biodiesel production; however, there is a requirement for improvement in the biosynthesis of triacylglycerides (TAG), critical engineering developments, improvements in mass culture, and further advances in downstream processing, including harvesting and extraction technologies. Currently, the high cost of obtaining biodiesel from microalgae limits commercial development. The estimated cost of biodiesel in photobioreactors with an oil content of 60% is US$3.96–10.56/L, whereas biodiesel from soybeans is tenfold cheaper [6]. Downstream processing of microalgal biomass (harvesting and product extraction) accounts for 70–80% of the total cost and has the most weight in terms of energy consumption [3]. Despite the challenges in producing low value commodities such as biodiesel from microalgae, a few strains have been commercially exploited for the production of animal feeds and high value products (>US$10,000/t) [7], namely, nutraceuticals and cosmeceuticals for human markets including polyunsaturated fatty acids (PUFAs) such as eicosapentaenoic acid (EPA) and docosahexaenoic acid (DHA); carotenoids such as astaxanthin and β-carotene, and phycobiliproteins such as phycocyanin and phycoerythrin. The potential for more diverse microalgal-derived products in recent years has grown rapidly and existing markets have received increased competition.

Microalgal products can be extracted using two processes; dry and wet routes. The dry route involves either spray drying, drum drying, freeze drying, or sun drying prior to extraction. The wet route is a process where the harvested biomass is disrupted to release the intracellular products. The dry processing method for biodiesel production requires 107.3 MJ/kg energy whereas the wet method comparatively uses 42.3 MJ/kg energy (84% of the energy requirement) [8]. It is believed that there is scope to decrease the energy consumption for extraction and transesterification (TE) to as low as 17 MJ/kg through optimisation of the overall wet extraction process [8]. Wet extraction without the need for lyophilisation of cells is therefore more desirable for energy-efficient extraction of intracellular products from microalgae.

Microalgal cell walls often contain algaenan and are notoriously difficult to lyse, often being resistant to chemicals and weak acids/bases [9]. *Nannochloropsis* and *Chlorella* strains cells are small (1–2 µm) and spherical and their cell walls are especially difficult to disrupt. *Haematococcus* cell walls contain a thick sporopollenin cell wall which even hinders acetolysis [10,11]. To date, physico-chemical methods are widely employed for the isolation of intracellular microalgal products. However, they are usually time-consuming and unless carefully controlled, are liable to cause degradation or unwanted chemical changes to the products, particularly carotenoids [12]. Due to the varying structural properties of different biochemical components, the identification of an optimal extraction solvent and technology to extract desired products remains a major challenge in microalgal biotechnology. Microwave-assisted extraction (MAE) offers an alternative green method for cell disruption and extraction of compounds from microalgae [13,14]. It has been critically evaluated for industrial-scale applications, revealing effective cell wall disruption with relatively low energy input, a rapid treatment time and the avoidance of the utilisation of hazardous substances [15].

In this review, we will be focusing on the benefits and challenges of MAE for obtaining microalgal products including lipids, pigments, vitamins, carbohydrates and proteins as individual compounds and/or as part of a biorefinery approach.

## 2. Current Extraction Methods for Biofuels and High Value Products from Microalgae

**Figure 1:**
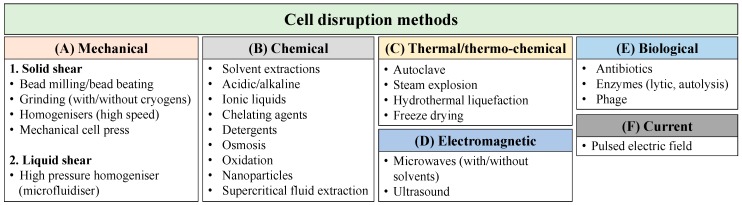
Summary of cell disruption methods used for microalgae.

The sustainability of biofuel production and high value products largely depends upon efficient extraction of the biochemical components. An ideal extraction method should be more selective towards extraction of specific microalgal products and simultaneously minimise the co-extraction of contaminants. Several methods have been used for extracting biochemical components from microalgae, whereby the initial stage is to disrupt the microalgal cell wall with the appropriate extraction method primarily depending on the rigidity of the cell wall and compounds of interest [16]. Broadly, the extraction methods are categorised as mechanical, chemical, thermal/thermo-chemical, electromagnetic, biological and current (Figure 1) and each have associated advantages and disadvantages (Table 1).

Commonly employed traditional solvent extraction techniques are labour intensive, utilise high quantities of toxic organic solvents, can expose bioactives to excessive light, heat and oxygen, and can result in changes in stereochemistry [17]. For solvent extraction of microalgal lipids, it has been determined that chloroform-methanol (1:1) resulted in the highest yields when comparing seven solvents and four microalgal species [18]. However, the use of toxic solvents such as chloroform and methanol on an industrial-scale will have a great impact on the environment and will pose a risk to human health. Other traditional methods such as grinding and cryogenic grinding with liquid nitrogen are reported to be extremely efficient but they are expensive and impractical for industrial applications.

Among mechanical methods, bead milling has been reported as the most effective method for cell disruption amongst nine different methods tested on two microalgal species with an optimal bead size of 0.5 mm [16]. However, it has been noted to be ineffective for the extraction of lipids from the Chlorophyte, *Chlorella vulgaris* [31]. Bead beating is often used in laboratories and agitated beads are often used for large-scale applications [32]. Nevertheless, overheating is an issue (also noted for homogenisers) and scale-up is often not economical [31]. Furthermore, beads add complexity to the system as it requires further separation [32]. Mechanical cell presses have been investigated for extraction of lipids from plants such as soybeans but due to the small size of microalgae, they often pass through without being disrupted [31]. Homogenisation and autoclaving have been suggested and have varying degrees of success, but both are considered impractical for large-scale applications [32]. Soxhlet is probably the most utilised method for the extraction of fats and oils from food matrices [33] and nutraceuticals from plant matrices [34]. However, the method is time consuming (~15 h), requires a high volume of toxic solvents [12], and later reported much lower yields of microalgal lipids when compared to other conventional extraction techniques [35].

To mitigate the use of toxic solvents, other chemical and biological methods have been developed as an alternative but often contaminate the cell extract and can create artefacts in the downstream analysis [36]. Biological methods with enzymes such as xylanases, pectinases or cellulases are constrained by economics with enzymes being relatively expensive and sometimes lacking efficiency [32,37]. Various novel physical techniques have been used in order to increase the efficiency of extraction and these include: supercritical fluid extraction [25], ultrasound assisted extraction [12,38], microwave-assisted extraction [12,14,39], pressurized liquid extraction [14,40] and pulse electric field lysis (PEF) [27]. Ultrasound operates in the range of 20–100 MHz and results in cavitation bubbles that expand and cause the cell wall to rupture [41]. Compared to horn sonication, bath sonication has been determined to be more commercially suitable as it only requires a single unit and has a lower power input [15]. Supercritical fluid extraction (SFE) has been suggested as a green technology where carbon dioxide under supercritical conditions is used as a non-toxic extractant in order to separate lipids from cells [13]. Similarly, accelerated solvent extraction (ASE), also known as pressurised liquid extraction (PLE) or pressurised solvent extraction (PSE) is widely applied for extraction of polar and non-polar lipids in corn and oat with various solvents [42] and for bioactive compounds such as antioxidants from two microalgal species, namely, *Dunaliella salina* and *Arthrospira platensis* [43]. However, both SFE and ASE are not suitable for scale-up due to high power consumption required for maintenance of high temperature and pressure [35]. Furthermore, a yield of thermo-labile components (such as carotenoids) could be severely compromised due to use of higher temperatures [44]. Pulse electric field lysis (PEF) has also been investigated for the extraction of desired products from microalgae whereby cells are exposed to brief pulses of a strong electric field as in electroporation for the transfer of DNA. Under optimal conditions, electric pulses temporarily cause the pores of the cell wall to open and the chemical contents are released as a form of ‘milking’ of the cells [27,45]. This method is currently constrained by the essential requirement of organic solvents for the extraction of carotenoids [46].

Current methods of extraction are hindered by extraction time, large solvent requirements, high energy inputs, and costly production processes with difficulties in scaling-up [47]. The use of microwaves for the extraction of microalgal compounds has been published frequently since 2008; primarily for lipids but also for other biochemical components including pigments, proteins, and carbohydrates. Microwaves offer the benefit of quick heating in comparison to conventional heating, selective energy dissipation and offer the same direction heat and mass transfer. Microwave extraction cuts down working times and often increases the yield and purity of the extract [12]. Microwaves were reported to result in the highest degree of cell disruption in *Nannochloropsis oculata* (94.92%) [21]. Overall, MAE offers an environmentally friendly option with reduced solvent requirements and appears to have real viability for the extraction of bioactives, which will be explored in this review.

## 3. MAE: Introduction and Working Principle

The use of microwave (MW) dielectric heating in analytical laboratories began in the late 1970s. Microwave energy was first described for extraction in 1986 [48]. MAE generates high frequency waves (ranging from 300 MHz (100 cm) to 300 GHz (0.1 cm)) with wavelengths of 0.001–1 m [32]. Microwave chemistry for extraction is where microwave radiation is applied at a frequency near 2.45 GHz (12 cm), causing dielectric heating primarily by absorption of the energy in water and other polar compounds available in wet biomass or a given sample [49].

Microwave heating results from the dissipation of electromagnetic waves in the irradiated medium. The dielectric properties and the average electric field affect the dissipating power. Microwaves cause the vibration of water and other polar molecules within wet biomass, thereby resulting in temperature increases in the intracellular liquids which subsequently causes the water to evaporate and exert pressure on the cell walls leading to cell disruption [50]. In addition, MWs disrupt hydrogen bonds and initiate the migration of dissolved ions, facilitating increased penetration of solvent into the sample [49]. The higher dielectric constant of water ensures that thermal energy is transferred to the cell walls more efficiently with microwave heating [21]. Unlike conventional heating, microwave heating is not constrained by thermal conduction or convection currents, thereby enabling a faster temperature increase [51]. The maximum temperature of the material heated by microwaves is dependent on the rate of heat loss and power applied [51].

There are two major types of microwaves; closed and open vessels. Closed vessel systems rely on controlled temperature and pressure, whereas in an open vessel system, only the part of the extraction vessel containing the sample is focused for microwave irradiation [52]. Recently, solvent-free microwave hydrodistillation (SFME) was adopted for laboratory-scale applications for the extraction of essential oils from different plants and fruits as an environmentally friendly and sustainable alternative [51] but to date, this has not been applied for microalgal biotechnology. SFME uses microwave heating and distillation at atmospheric pressure, where less CO_2_ is emitted into the atmosphere (200 g CO_2_/g of essential oil compared to traditional methods emitting 3600 g CO_2_/g of essential oil) [53]. Microwave hydrodiffusion and gravity (MHG) combines microwave heating and earth gravity at atmospheric pressure [54] allowing the extraction of essential oils without distillation and evaporation that are the most energy consuming processes between the unit operations [55]. There are several key operational factors determining the efficiency of MW extraction such as the species of microalga, power of the MW, temperature, and solvent properties and volumes used [32].

## 4. MAE of Biochemical Components from Microalgae

Microwaves have been used for obtaining a variety of bioactives from a range of microalgal classes including the Chlorophyceae, Bacillariophyceae, Eustigmatophyceae and Phaeophyceae. The majority of work evaluating the effectiveness of microwaves on extraction has been conducted on lipids followed by pigments, whereas few publications exist on the extraction of carbohydrates, proteins and other compounds of interest such as vitamins.

### 4.1. Lipids

#### 4.1.1. Pre-Treatment: Lipids as a Feedstock for Biodiesel

A variety of solvents have been investigated for lipid extraction with dry and wet methods using MWs, including hexane, chloroform:methanol (different ratios), and ethanol (Table 2). The ratio of solvent to sample ranged from 1:1 to 400:1 with hexane requiring less volume and chloroform:methanol requiring much higher volumes (Table 2). Different concentrations of starting microalgal biomass were also tested ranging up to 5 g/L dry weight (DW), where higher concentrations of feedstock appeared to have little effect on overall extraction efficiency. The MW operating conditions varied from 45–140 °C, with pressures ranging from 1–80 bar and extraction times of 2.5 min to 60 min (Table 2). To date, only one study has been conducted on comparing dry vs. wet extraction for microalgal lipids using MWs, where the dry method resulted in a 18.95% increase in lipid yield for *Scenedesmus obliquus* [56].

Hexane is commonly utilised for extraction processes as it is often threefold cheaper than other non-polar solvents such as cyclohexane, easy to recover after extraction (with up to 95% solvent recovery), and has high selectivity for neutral lipids [6,35,47]. MAE of lipids using hexane (wet method) from *Nannochloropsis salina* only requires 9.89 MJ/kg energy for 24.3% DW fatty acid methyl esters (FAMEs) [57]; whereas chloroform:methanol extraction from *Chlorella* requires 25.2 MJ/kg energy for 18.7% DW lipid [58]. In addition, MAE with hexane can result in a higher quality oil with higher recoveries for unsaturated and essential fatty acids (FAs) where more than 77% of recoverable oil is extracted in 30 min compared to only 47% using conventional heating [47]. Conversely, it has been determined that [59] extraction method (chloroform:methanol (2:1)) results in a 44.4% increase in biodiesel yield compared to hexane [60]. However, both the solvents are toxic, non-environmentally friendly, pose a health risk to humans and are expensive. Many researchers have directed their approach towards the use of non-chlorinated solvents which are much more economical, less toxic and have a reduced environmental impact compared to chlorinated solvents and more importantly, they have similar lipid extraction efficiencies as that of chlorinated solvents. However, there is no firm evidence and agreement on the use of a single non-chlorinated solvent system for microalgal lipid extraction as there were mixed conclusions reported by various studies in the past literature [16].

Biodiesel (in the form of 40% methyl soyate in ethanol) has been used for lipid extraction from *Nannochloropsis* sp., resulting in the highest lipid yield to date (56.6% DW), 6.80% higher than using chloroform:ethanol [61]. A high-power input was used (1200 W) with a higher temperature (120 °C) and timespan (50 min); however, the energy input required for this process was not specified [61]. Another promising environmentally friendly method is using protonic ionic liquids which resulted in moderate levels of cell disruption for *Chlorella* sp. (74.75%) and *Chlorococcum* sp. (70.03%) in a wet extraction process, but the yields of lipid were low (3.5 and 0.8% DW respectively) [30]. Using hydrogen sulphate ionic liquids in combination with 2% HCl resulted in higher lipid yield (27% DW) for *Chlorella sorokiniana* [62].

The lipid and biodiesel yield is strongly dependent on the species of microalga investigated along with the MW operating parameters. *C. vulgaris* and *Nannochloropsis* sp. were investigated in several studies (Table 2) and consistently attained higher lipid contents (maximum content 31.7 and 38.31% DW, respectively). High lipid recoveries were generally observed with high wattages (>1000 W) and temperatures (>65 °C); however, the effect of the timespan on lipid extraction remains to be elucidated. Furthermore, the use of different solvent ratios within the same solvent system has a large effect on extraction efficiency. Therefore, it is essential to carefully select the optimal extraction solvent system and their ratios prior to their comparison with the conventional solvent systems. The selected solvent system for lipid extraction should also be evaluated for their selectivity towards desired lipid classes of interest with a minimal extraction of non-lipid contaminants. More direct comparisons are needed between different solvent systems for a variety of species and MW operating parameters (wattage, temperature and timespan) with an emphasis on wet processing, as this is better suited for sustainable large-scale production.

#### 4.1.2. Direct Transesterification for Biodiesel Production

Biodiesel is safe, renewable, non-toxic, and biodegradable in water (98% biodegrades in just a few weeks), containing fewer sulphur compounds than diesel [41]. For the formation of biodiesel, lipids need to be transesterified. TE involves the reaction of an oil with an alcohol (often in the presence of a catalyst) to form fatty acid esters and glycerol as a co-product. Triglycerides are converted to diglycerides and then subsequently to lower glycerides with one glyceride yielding one FAME molecule [41]. Microwaves have so far been successfully used for the extraction of lipids from microalgae, but they can also be used for direct transesterification (DT) (Table 3), where extraction and TE are performed in a single step [65,77]. High biodiesel yields can be obtained when using sodium (99.3% conversion) or potassium methoxide (98.5% conversion) as catalysts [41]. However, TE uses high volumes of methanol along with an acid or base and operates under high temperatures.

For biodiesel production, DT using MWs is desirable to reduce processing time and the overall cost of the process; however, fewer publications exist on this approach. To date, the highest biodiesel conversion efficiency using this approach (80.13%) has been obtained for *Nannochloropsis* sp. using methanol as an extractant and potassium hydroxide as a catalyst at 60–64 °C within 6 min, but this required a high level of energy (127 MJ/kg) [82]. However, there is a desire for a more environmentally friendly solvent/solvent free process with reductions in energy input.

#### 4.1.3. High Value Lipids: EPA and DHA

The omega-3 FAs, EPA and DHA, are of interest in industrial markets with DHA being commercially produced for incorporation into infant formula milk [83]. Cravotto and co-workers [12] obtained a 17.8% oil yield when using hexane as an extractant with MWs compared to Soxhlet, which only yielded 4.8% DW. However, the DHA yield was not reported. Using double sonication and Soxhlet extraction, the fatty acids were found to be comprised of 39.3 and 39.5% DW DHA, respectively; hence, there is scope for extraction from *Crypthecodinium cohnii* using MWs, but this remains to be elucidated. EPA has been obtained from *Nannochloropsis gaditana* (1.18% DW) using methanol as an extractant at 30–35 W, 90 °C with a heating time of 10 min (Table 2) [66]. EPA has been observed to occur in *Phaeodactylum tricornutum* up to 4.9% DW [84] and this may offer a more suitable organism for economic recovery.

### 4.2. High Value Pigments

Pigments in microalgae encompass chlorophylls and carotenoids. Carotenoids are a family of more than 600 naturally occurring pigments synthesised by higher plants, algae, fungi, and bacteria. Two major groups of carotenoids have been characterised on the basis of their chemical structure; the carotenes (composed of carbon and hydrogen) and the xanthophylls (oxygenated derivatives). In nature, it has been observed that most carotenoids are of the *trans* form and the *cis*-isomers are known to be thermodynamically less stable than *trans* isomers [32]. When dealing with pigments it is essential to develop a method that does not result in the degradation of pigments or induces changes in the stereochemistry. Carotenoids are vulnerable to light, heat and oxygen [85], and light and air can result in a synergistic breakdown of all-*trans* and *cis*-isomers [86]. Microwaves operating at 600 W have been observed to convert *trans*-astaxanthin isomers to *cis*-isomers [87]. It was not known what temperature the microwave reached and it could be that the temperature led to degradation, as carotenoids are known to degrade at temperatures above 60 °C [88]. It has been reported that temperatures above 75 °C do not increase astaxanthin recovery with microwave technology [89]. Although microwaving caused an isomer change, ultrasound was found to degrade pigments into colourless compounds [87].

Pigment extraction from microalgae, especially *Chlorella* and *Haematococcus*, is notoriously difficult due to their cell wall rigidity [9]. MAE can achieve the same yields as hot soaking but in minutes rather than timeframes of 30–60 min [88]. It has been suggested that MAE is effective for obtaining pigments from microalgae which have high mechanical resistance such as diatoms with silica frustules inducing frustules permeabilisation without causing rupturing of the cell [88]. For those species that lack a frustule and thick outer exopolysaccharide envelope, conventional techniques are more suitable in terms of yield and extraction time [88]. To date, chlorophyll-a, chlorophyll-b, astaxanthin, β-carotene and fucoxanthin have been extracted from microalgae (Table 4). With respect to solvents being used for pigment extraction, hexane has been found to be effective for lipid extraction but is poor for polar pigments such as fucoxanthin as hexane is non-polar [40]. Ethanol can be an alternative to hexane as it is non-toxic [6]. However, acetone has been observed to result in the higher extraction efficiency for astaxanthin which has been attributed to its lower polarity [89].

There has been a particular focus on astaxanthin extraction from *Haematococcus pluvialis* as it has a high selling price of up to US$7000/kg [92] but currently, the cost of production is high (US$3000–3600/kg) [93]. For astaxanthin, each double bond from the polyene chain has been found to exist in two different configurations as geometric isomers *cis* or *trans*. The *trans* astaxanthin in *Haematococcus* is 81.4% of the isomers [94]. It has been determined that astaxanthin may present three configurational isomers of the *trans* form (3R, 3′R; 3R, 3′S and 3S, 3′S) with the 3S, 3′S form being the most abundant astaxanthin isomer in nature and in the microalga *H. pluvialis* and the preferred enantiomer for pigmentation of salmonids in aquaculture [95]. The 3S, 3′S isomer has also been reported to be the most beneficial for human health [96,97]. Mechanical treatment with a homogeniser has resulted in a maximum astaxanthin yield of 1.8% DW with various extraction methods investigated; however, MAE was excluded [11]. For MAE of astaxanthin, the highest yield to date has been 0.80% DW with only 37% recovery using acetic ether at a low temperature (45 °C) [38]. This is a low yield considering astaxanthin is 4% DW of the cells [98]. Using acetone, a 74% astaxanthin recovery could be achieved [89], but the initial content of the starting biomass was not reported. Further work needs to be conducted on obtaining higher recoveries of astaxanthin with a focus on the isomers formed after MAE. It is also possible to exploit the complex life-cycle of *H. pluvialis* and produce astaxanthin in the motile morphotype that lacks the thick wall of aplanospores [99] and therefore offers higher potential of astaxanthin recovery through MAE.

Another high value pigment of interest is fucoxanthin which can reach US$150/g when sold in capsule form [100] due to its anti-obesity, anticancer, anti-inflammatory and anti-diabetic properties [101]. The highest yields obtained to date were from *P. tricornutum* using PLE at 100 °C for 10 min (1.63% DW) [40]. With MAE, the highest yields attained have only been 0.46% DW fucoxanthin [14] but this was using a low extraction time (2 min) and a low ratio of solvent:sample (20:1). This is the first study on the extraction of fucoxanthin and further work needs to be conducted as demand is likely to rise. Low yields of other pigments have also been reported for chlorophylls and β-carotene with the maximum yield of β-carotene (0.12% DW) using acetone [88]. Careful optimisation of MAE of pigments is required, ensuring that they are not degraded or undergo isomer changes. Moreover, a thorough optimisation of MAE parameters is required with a focus on using other environmentally friendly and economical solvents for increasing the yield of pigments with particular attention on the energy inputs.

### 4.3. High Value Proteins, Vitamins, Carbohydrates and Others

In the past few years, other compounds have been extracted from microalgae using MAE such as proteins (phycoerythrin, phycocyanin, allophycocyanin), vitamins and bulk products (carbohydrates and methane) (Table 5). The phycobiliproteins, phycoerythrin, phycocyanin and allophycocyanin are used for food pigmentation and as fluorescent dyes in research. Depending on their applications they can range from US$130–30,000/kg [102,103]. Typically, phycobiliproteins are extracted from microalgae using a freeze-thaw process or hot soaking [104]. Recently, methods without the use of solvents have been investigated using MW technology for efficient extraction (Table 5). Currently, *A. platensis* can produce phycocyanin up to 20% DW [105]. Using MAE it is possible to extract 0.23% DW phycocyanin from *A. platensis* using dry biomass (60 °C, 15 min) [91]. This is a low yield and may be attributed to the low power output (400 W), along with an inefficient solvent and low solvent to sample ratio (7:1) (Table 5). However, high power can result in the risk of thermal degradation/deterioration. Additional reports suggest that other thermolabile products (flavonoids) were not increased with the increase in power outputs of 500–1000 W [54]. A solvent-free approach resulted in yields of 3.48% DW phycocyanin at 100 °C from *Porphyridium purpureum* biomass (dry) [104]. *A. platensis* is also a valuable source of allophycocyanin with up to 19.8% DW [103]. To date, MAE of allophycocyanin from *A. platensis* has not been explored but yields of 3.51% DW have been obtained in *P. purporeum* using a dry method [104]. For phycoerythrin production, *P. purporeum* has been identified as a good source (3.3% DW) [106] where MAE resulted in a 3.48% DW yield at 40 °C in 10 s, whereas conventional soaking at the same temperature required 60 min for a comparable yield [104]. In MAE it was observed that increasing the time had no further improvements in yields of phycoerythrin, whereas an increase in temperature (50–100 °C) resulted in decreased yields, confirming thermal damage occurs above 40 °C.

Other products of interest extracted from microalgae using MAE are vitamins. Vitamins are essential micronutrients that cannot be synthesized de novo and must be obtained from the diet. Vitamin C (ascorbic acid) and vitamin E (tocopherols) are used as food additives with antioxidant potential [108]. α-tocopherol was obtained from *A. platensis* at 400 W, 40 °C when extracted for 50 min but the yield was very low (<0.01% DW) [91]. B vitamins are eight essential nutrients that are essential for growth and reproduction, but their intakes are frequently lower than the daily recommendations. Microalgae offer a non-chemical, vegan source of B vitamins as most people obtain B vitamins from cow’s milk and can also be used as a natural source to fortify milk for non-vegans. Two essential B vitamins; vitamin B1 (thiamine) and vitamin B2 (riboflavin) have been identified in microalgae. Vitamin B1 is incorporated in food to treat and prevent thiamine deficiency and those disorders that result from it such as beriberi; vitamin B2 is used to prevent and treat riboflavin deficiency and has been reported to prevent migraines [109]. Thiamine (0.085% DW) and riboflavin (0.010% DW) were obtained from *A. platensis* but in low yields [91]. Both are water-soluble vitamins and their extraction efficiency increased with the water volume percentage in the solvent. Solvent ratio and extraction time were found to be critical parameters affecting extraction using MWs. Currently, these yields of vitamins are low and further operating parameters of MAE need to be optimised in order to improve the extraction efficiency.

Lastly, from the residual biomass subsequent to lipid extraction, methane can be obtained. To date, using microalgal pre-treatment the highest yield of methane obtained was 307.11 mL methane/g total volatile solids [107]. This yield was 2.4-fold higher than that in a later study by [29]; however, the energy consumption was increased by 1.9-fold (65.4 MJ/kg). Soluble carbohydrates can also be obtained as bulk products from microalgae, but they have not yet been characterised [29]. They have the potential to be used as a feedstock for bioethanol, but energy requirements have to be taken into account. For the extraction of carbohydrates using MWs only, the energy required is 34.3 MJ/kg [29], which is more than that required for lipids (1.18 MJ/kg).

## 5. MAE vs. Current Extraction Methods

A wide range of cell disruption techniques has been reported for efficient extraction of bioactives from microalgae. MAE has been found to outperform other extraction methods in several comparison studies with a mixed species culture and monocultures of *Botryococcus*, *C. vulgaris*, *Scenedesmus* and *Dunaliella tertiolecta* for lipid extraction [31,60,71,75] and for EPA production from *N. gaditana* [66]. However, comparative studies for pigment extractions omitted MAE, such as astaxanthin from *H. pluvialis* [110] and fucoxanthin from *P. tricornutum* [40].

MAE has several advantages over conventional extraction methods for cell disruption as a pre-treatment method and for DT methods from lipids. In addition to the excellent recovery of compounds of interest, other key advantages include a single step conversion process, short reaction times, reduced solvent usage, and removal of water (expensive step) is not required; instead, water serves as an excellent solvent for extraction and is non-toxic [8]. In terms of extraction times, soxhlet extraction can take up to 15 hours whereas MAE can only take a few minutes with ten times less solvent for oil extraction [12]. In terms of energy consumption, high pressure homogenisation is the most energy intensive process (529 MJ/kg) for biofuel extraction, whereas microwaving and hydrodynamic cavitation were observed to require the least amount of energy (9.6 and 33 MJ/kg, respectively) [8]. MAE and UAE are considered to be the most economically viable options for extraction of lipids and pigments due to their high degree of efficiency for cell disruption, high yields of bioactives, and rapid extraction rates [21]. UAE has been identified as energy intensive in comparison to MAE, suffering from steep thermal gradients, and has been observed to be insufficient for cell lysis against some microalgal cells such as the diatom *Cylindrotheca closterium* [88,111].

MAE has been widely investigated for the extraction of lipids; however, reports on the extraction of pigments and other high value products are just starting to appear in the literature and are very few in number. There are difficulties in comparing the literature for extraction efficiency as there are biological (wet/dry, concentration, species and strain) and operational (solvents and microwave settings) variabilities, which need to be kept constant for efficient comparison between different extraction techniques.

## 6. Recent Trends and Developments: MAE for Microalgae

At a laboratory-scale, MAE has been primarily applied for extraction of individual product components from microalgae (primarily lipids and carotenoids). In order to reduce the cost of the overall process, a transition from a dry to wet method is desirable [112]. Moreover, for biodiesel production, DT rather than a separate extraction and TE process has led to reductions in energy input overall extraction cost [82]. To date, MAE has mainly focused on the extraction of lipids; however, the feasibility of a biorefinery using MAE for the production of lipids, carbohydrates and proteins has been recently investigated [56].

With advances in laboratory-scale MW technology, there have been a few reports of industrial-scale application for the extraction of oil from soybeans and rice bran [113] and volatile and non-volatile organic compounds from baldo leaves (*Peumus boldus*) [114]. Similarly, the potential of MAE for industrial-scale applications in microalgal biotechnology has been discussed in detail [31]; however, reactor designs need to be developed for sustainable implementation [22]. The cost of maintenance was also highlighted as a bottleneck for upscaling [115] which needs to be addressed. A continuous MAE system has been developed for the rapid extraction of oil from microalgae at a laboratory-scale [47]; however, this was not followed up for large-scale application and requires thorough evaluation of the energy input and overall cost. To date, only one report has extensively compared the industrial-scale application of different extraction techniques for microalgae, where the authors employed a variety of parameters for evaluation and concluded MAE as a moderately suitable technique for scale-up [15]. Overall, as the capital expenditure is high for MAE, the microalgal biorefinery concept needs to be in place in order to offset this cost, but this has not been showcased to date.

## 7. Challenges Involved in MAE

Although microwaves have been successful to date for obtaining a variety of products, for future process development there are several constraints that need to be addressed. There is a safety concern to consider when using microwaves as they operate at high temperatures and in conjunction with solvents can pose a high risk to the operator [41]. Closed vessel MW systems pose a high risk of injury to analysts due to the use of higher pressures, whereas polytetrafluoroethylene (PTFE) material used in construction does not allow the use of high temperature and the additional cooling step is required to avoid the loss of extracted volatile components [52]. Comparatively, open vessel systems are safer than closed vessel systems but the extraction conditions are less reproducible and many samples cannot be processed simultaneously, requiring longer extraction times to achieve efficiencies similar to that of closed vessel systems [52].

A major challenge with MWs, particularly domestic MWs is the uneven distribution of radiation within the cavity and consequently, energy is not homogeneously dissipated resulting in uneven heating of the sample [116]. The major concerns with MAE is that it is limited to polar solvents and is not suitable for volatile target compounds [26,117]. Once the microalgal extracts have been disrupted and the intracellular contents have been released, an additional separation process is required to remove solid residues [49]. There is a mixture of studies on dry and wet biomass and it is well known that it is more expensive to process dry microalgal material for extraction [8]. For a given product of interest, thorough investigations are required comparing the efficiency of the extraction from the dry and wet material. To date, only one study investigated the extraction efficiency of lipids from *Scenedesmus* and it was concluded that dry extraction only results in a 16.5% increase in lipid yield [76]. In MAE studies, there is a lack of consistency for the extraction of bioactives of interest with different wattages (30–1400 W), pressures (1–300 bar) and timespans (from 50 s to 50 min) being used for a given species, extractant and concentration/state of the biomass. In addition, there are large variations in the energy consumption for MAE of various bioactives, ranging from 1.2–140.8 MJ/kg for lipid (Table 2) and 34.3 MJ/kg for protein and carbohydrates [29].

Selection of appropriate extraction solvent/solvents and solvent ratios presents a major challenge in MAE and is mainly driven by factors such as selectivity of solvent/solvents towards analyte of interest, the property of solvent to absorb microwaves, the interaction between solvent and matrix and compatibility with subsequent analytical platform employed. From a biofuels perspective, the commonly applied MAE protocols use solvent mixtures which include chloroform and methanol due to their excellent ability to penetrate the cell wall as they have high polarity index values, suggesting higher solubility for all polar lipid compounds. However, both the solvents are toxic, non-environment-friendly, pose a health risk to humans and are expensive. Furthermore, the use of different solvent ratios within the same solvent system has a large effect on extraction efficiency and requires careful optimisation. In MAE, high volumes of solvent are often used in relation to the sample: chloroform:methanol (100:1) used for the extraction of lipid from *C. vulgaris* and *D. tertiolecta* [60,71], acetone (100:1) and acetic ether (100:1) for the extraction of astaxanthin from *H. pluvialis* [38,89]. Overall, all the above constraints represent a major challenge and require careful optimisation in order to explore the full potential of MAE for extraction of commercially viable products from microalgae.

## 8. Future Prospects: MAE as a Cost Effective Biorefinery Approach

**Figure 2:**
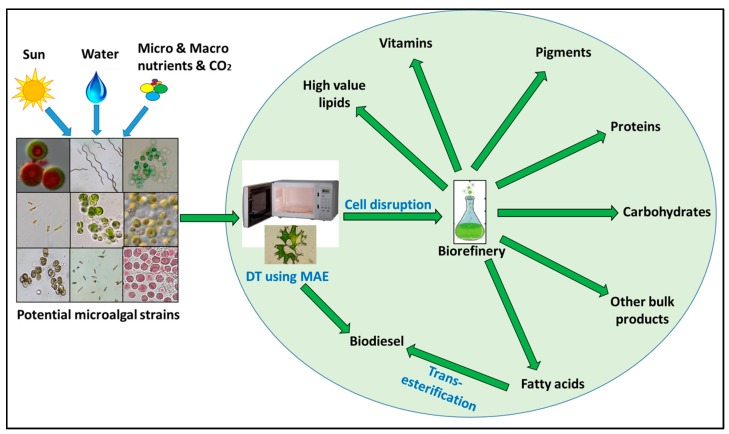
Overview of MAE based biorefinery for microalgal biotechnology.

Overall, a wide range of products has been obtained from microalgae using MAE. Lipid extraction (as a precursor for biodiesel) using MAE has potential but requires further optimisation of its process parameters to make the overall process economically sustainable. Although traditionally used at laboratory-scale, MAE has the potential to be upgraded to a commercial scale as demonstrated recently for the production of biodiesel from waste cooking oil [52]. Additionally, it has been revealed that the most promise lies in obtaining biodiesel through DT using MWs, which remains to be explored at an industrial-scale. Isopropanol/hexane has been suggested as the most appropriate solvent for the extraction of proteins and carbohydrates from lipid extracted algae (LEA) biomass [76] but this is unsuitable for a large-scale application. To date, most commercial processes have focused on a single product approach but there have been recent reports on utilising microalgae as a biorefinery and producing multiple products (Figure 2). A biorefinery aims to replace oil with biomass as a feedstock for bioenergy and a spectrum of marketable products utilising different biochemical fractions including lipid, protein, vitamins, carbohydrate and pigments through a sustainable process [6,117]. To date, only Ansari and co-workers [56] focused on a biorefinery approach, whereby lipids were extracted from *S. obliquus* using MWs, whereas different extraction methods were employed for proteins and carbohydrates. Proteins were deemed the most costly biochemical fraction, followed by lipids and carbohydrates. Carotenoids were omitted from this study. For a successful biorefinery, it was determined that proteins should be extracted first, followed by the extraction of lipids and carbohydrates. However, to date, MAE for industrial-scale applications has not been investigated thoroughly and currently is only suitable for laboratory-scale applications.

In developing the biorefinery platform, more emphasis should be on selecting the correct organism with the most commercial potential. Biofuels from microalgae have been investigated since the 1970s with The Aquatic Species Programme (ASP) revealing that biodiesel had the greatest potential of all biofuels [118]. Nevertheless, biodiesel from microalgae was not and is not cost competitive with oil refinery platforms. Oil production from microalgae in the optimal scenario is US$1.65/L [119] but the current price of Brent crude oil is ~US$50/barrel, equating to US$0.26/L. Therefore, in order to create an economically sustainable biorefinery platform, an involvement of high value products is required.

Currently, there are three microalgal species that have been investigated for producing a suite of products that have commercial potential; *P. tricornutum* for EPA, chrysolaminarin (carbohydrate) and fucoxanthin [120], *P. purpureum* for EPA, zeaxanthin, β-carotene, exopolysaccharides and phycobiliproteins [121] and *Nannochloropsis* sp. for EPA and high value proteins [122]. Due to the higher lipid content and greater biodiesel conversion efficiency of *Nannochloropsis* sp., there is a greater potential for establishing the economically sustainable microalgal biorefinery using MAE, where biodiesel can be produced in conjunction with EPA, pigments and phycobiliproteins. Phycobiliproteins have been successfully extracted from *P. purporeum* and thus through process development, this can be applied to *Nannochloropsis*. However, further work and a detailed life cycle analysis are warranted.

MW technology offers great potential for a biorefinery approach provided the challenges can be addressed with an emphasis on using green solvents and reducing the energy input. Alternatively, a ‘milking’ process where operating parameters are selected which temporarily open pores within the cell wall (as in electroporation) holds promise and can be a viable option for microalgal biotechnology as postulated by [58]. Temporarily opening the pores would release biochemical components into the media, which would result in a simplified extraction process with a reduction in energy requirement and without killing the cells.

## Figures and Tables

**Table 1:** Advantages and disadvantages of current cell disruption techniques for microalgal biotechnology.

Method	Operates at Industrial Scale	Suitability for Commercial Application	Advantages	Disadvantages	Ref.
High pressure homogeniser	✓	-	Destruction of cell walls at room temperature, effective for neutral lipid extraction	High energy input, not effective for extraction of high molecular weight proteins	[15,19]
Mechanical cell press	✓	-	Industry standard for oil recovery from oilseeds	Inefficient cell disruption, high energy input	[20]
Hydrodynamic cavitation	✓	-	Relatively low energy input	Cavitation area limited	[21]
Horn sonication	✓	++	Effective cell wall disruption, low maintenance cost, relatively rapid process, hazardous chemicals are not required	Multiple units required, cavitation area limited, high operational costs and energy input	[15]
Bath sonication	x	+++	Effective cell wall disruption, minimal maintenance cost, relatively rapid, no hazardous substances required	High operational costs and energy input	[15]
Microwaves	x	++++	Effective cell wall disruption and excellent recovery of bioactives, relatively low energy input, fast heating and short reaction time, reduced solvent usage	Generates heat, high maintenance cost	[15]
Bead milling/beat beating	✓	++	Effective cell wall disruption, rapid extraction	Varied efficiency across species, additional step required to remove beads, high maintenance costs and energy input	[15]
Osmotic shock	x	-	Low energy input, easier to scale-up	Inefficient cell disruption, generation of waste saltwater, time consuming	[22]
Acid/alkali	✓	-	Low energy input	Requires disposal of acid/alkali after extraction, carotenoid degradation	[22]
Enzymatic hydrolysis	✓	++	Effective cell wall hydrolysis, high selectivity, mild treatment, carotenoid bioactivity not affected	High cost of enzymes, longer treatment time, enzymes must be disposed of after use	[15]
Autoclave	x	+	Low maintenance cost	High energy input, not suitable for pigments	[11,23]
Steam explosion	✓	+++++	Effective cell wall disruption, low maintenance costs, relatively low energy input	Varied efficiency across species	[15]
Freeze drying	✓	+	Mild operating conditions, drying and extraction can be incorporated in one step, does not affect cellular components	Cell disruption variable and often the integrity of the cell wall is weakened but not disrupted, cost associated with pump maintenance, time consuming, expensive, high energy input	[15]
Nanoparticles	x	-	Non-toxic	Expensive, additional step required to remove nanoparticles, technology in its infancy	[24]
Supercritical fluid extraction	✓	+	Polarity of solvent is tunable, fast process, uses non-toxic solvents such CO_2_, effective for carotenoid extraction	Expensive, not suitable for scale-up	[13,25]
Grinding (with/without cryogens)	x	-	Quick and efficient at a laboratory-scale	Time consuming, degradation of some of the bioactives	[26]
Pulse electric field	✓	+	High selectivity, mild treatment, carotenoid bioactivity not affected, relatively low energy input	Still in its infancy	[27]
Hydrothermal liquefaction	x	-	Uses a wet feedstock	High variability in recovery, high energy input and temperature, requires expensive catalyst	[28,29]
Ionic liquids	x	-	Low cost	Still in their infancy, issues over toxicity	[30]
Soxhlet extraction	✓	+	Cost-effective, easy to scale-up	Long extraction time, uses large amounts of solvents (often toxic)	[12]

✓: Yes; x: No; -: Not suitable; +: Weak; ++: Moderate; +++: High; ++++: Higher; +++++: Very high.

**Table 2:** Lipids extracted from microalgae by MAE as a pre-treatment method.

Microalgal Strain	Dry/Wet Method	Solvents Used	Ratio (Solvent to Sample)	Volumes Added	Microwave Settings	Product & Yield	Energy Use (MJ/kg)	Ref.
*Crypthecodinium cohnii*	Dry	Hexane	18:1	2 g of milled algal powder, 35 mL hexane	2.45 GHz, 45 °C, 30 min	17.8% oil yield	x	[12]
*Botryococcus* sp.	Dry	Chloroform:methanol (1:1)	200:1	0.5 g of algae powder, 100 mL distilled water, 100 mL chloroform:methanol	2.45 GHz, 100 °C, 5 min	28.1% lipid	x	[31]
*Chlorella vulgaris*						10% lipid		
*Scenedesmus* sp.						10.4% lipid		
*Scenedesmus obliquus*	Wet	Hexane	1:1	Equal volume of hexane to sample after heating	2.45 GHz, 1200 W, 95 °C, 30 min (5 min intervals)	31.38% wet weight (77% recoverable oil)	x	[47]
*Chlorella* sp.	Dry	Chloroform:methanol (1:1)	400:1	0.5 g algae, 200 mL chloroform:methanol	2.45 GHz, 100 °C, 5 min	26 mg/g FAMEs	x	[63]
*Nostoc* sp.						19 mg/g FAMEs		
*Tolypothrix* sp.						21 mg/g FAMEs		
*Chlorella vulgaris*	Wet	Chloroform:methanol (1:1)	x	500 mL culture pelleted	2.45 GHz, 100 °C, 5 min	18.14% lipid	x	[26]
*Chlorella vulgaris*	x	Chloroform:methanol (1:1)	100:1	1 g algae, 100 mL chloroform:methanol	300 W, 50 °C, 30 min	31.9% DW lipid	x	[60]
*Chlorella vulgaris SAG 211-12*	Dry	Chloroform:methanol (1:1)	100:1	0.5 g algae, 50 mL distilled water, 50 mL chloroform:methanol	2.45 GHz, 1000 W, 2.5 min	9.59% DW lipid	x	[64]
*Chlorogleopsis fritcschii*	Dry	Dichloromethane	25:1	1 g algae, 10 mL deionised water, 25 mL dichloromethane	1200 W, 140 °C, 15 min	1.4% DW lipid	x	[28]
*Nannochloropsis oculata*						11.3% DW lipid		
*Pseudochoricystis ellipsoidea*						37.5% DW lipid		
*Chlorella PY-ZU1 GM*	Wet	Chloroform:methanol (1:1)	50:1	50 mL algal culture (1 g DW), 50 mL chloroform:methanol	80 °C, 10 min	18.7% DW lipid	25.2	[58]
*Nannochloropsis* sp.	Wet	Chloroform:ethanol (1:2)	45:1	3.3 g wet algal paste, 50 mL chloroform, 100 mL ethanol, 40 mL deionised water	2.45 GHz, 1200 W, 120 °C, 50 min (5 min ramp, 15 min hold, 30 min cool-down)	53% DW lipid	x	[61]
		40% methyl soyate in ethanol	x	3.3 g wet algal paste, 40% methyl soyate in ethanol		56.6% DW lipid		
*Phaeodactylum tricornutum*	Wet	Chloroform	x	45 mL culture pelleted, 5 mL distilled water, 3 mL chloroform	2.45 GHz, 1000 W, level 4, 90 °C, 5 min	32% (*w*/*w*) glycerides	x	[65]
*Chlorella vulgaris*						21% (*w*/*w*) glycerides		
*Chlamydomonas reinhardtii*						7% (*w*/*w*) glycerides		
*Scenedesmus dimorphus*	Wet	Chloroform:methanol (1:1)	40:1	200 mg wet algae, 8 mL solvent	557 W, 1 min then 254 W, 4 min	17.2% DW lipid	x	[23]
*Selanastrum minutum*						21% DW lipid		
*Chlorella protothecoides*						17% DW lipid		
*Nannochloropsis gaditana*	Dry	Methanol	x	5 g algae	2.45 GHz, 30–35 W, 90 °C, 10 min	14.82% DW FAs; 1.18% DW EPA	10.9 Wh/g FA	[66]
Unknown microalga	Dry	n-heptane:isopropanol (2:1)	x	5 g algae	1000 W, 40 min	28% DW lipid	x	[67]
Mixed culture of microalgae	Dry	Methanol:chloroform (1:1)	10:1	500 mg algae, 2.5 mL methanol, 2.5 mL chloroform, 1.25 mL 1.5% sodium sulphate, 1 mL deionised water	400 W, 100 °C, 5 min 30 s (70 s temperature ramp, 45 s hold, 3 cycles)	33.7% DW lipid	x	[68]
*Isochrysis* sp.	Dry	Methanol:chloroform (1:2)	x	0.5 g algae, 50 mL distilled water	2.45 GHz, 1200 W, 45 °C, 30 MPa, 5 min	7.8% DW FFAs; 0.08% DW EPA	x	[69]
*Nannochloropsis gaditana*						10.8% DW FFAs; 0.47% DW EPA		
*Scenedesmus almeriensis*						3.1% DW FFAs; 0.22% DW EPA		
*Tetraselmis* sp.						4.8% DW FFAs; 0.1% DW EPA		
*Scenedesmus* sp.	Dry	Chloroform:ethanol (1:1)	20:1	40 mL mixture chloroform:ethanol, 2 g DW algae	1000 W, 100 °C, 10 min	53% lipid	1.18	[39]
*Chlorella* sp.	Dry	Ethanol	12:1	4 g algae, 48 mL ethanol and 2% NaOH catalyst	700 W (50% power), 75–80 °C, 6 min	20.1% DW FAMEs	x	[70]
*Dunaliella tertiolecta*	Dry	Chloroform:methanol (2:1)	100:1	0.2 g algae, 20 mL chloroform:ethanol	490 W, 2 min 40 s	57.02% lipid recovery	x	[71]
					160 W, 7 min	56.98% lipid recovery		
Nannochloropsis salina	Wet	n-hexane (added after microwave extraction)	3:1	60 mL algal culture, 15 mL n-hexane (biomass loading 25%)	1400 W, 205 °C, 25 min, 21.5 bar	24.3% DW FAMEs; 1.65% DW EPA	9.89	[57]
*Nannochloropsis* sp. (BMRI)	Wet	Methanol:hexane (1:2)	x	10 mL algal culture	2.45 GHz, 1000 W (70% of power), 65 °C, 1 bar, 5 min	38.31% DW lipid	x	[72]
*Nannochloropsis oculata*	Wet	Ethanol:hexane (3:1) (added after microwaving)	23:1	4.3 g algae (1 g dry algae equivalent), 17 mL ethanol, 8 mL distilled water, 5.6 mL hexane	2.45 GHz, 1025 W (100% power), 5 min (15 s heating bursts and cooled for 15 min)	5.2% DW lipid	140.78	[21]
*Scenedesmus obliquus*	Dry	Chloroform:methanol (2:1)	20:1	1 g algae, 20 mL chloroform:methanol	1000 W, 100 °C, 10 min	21.43% DW lipid	x	[73]
*Stigeoclonium* sp.; *Monoraphidium* sp.; *Nitzschia* sp. & *Navicula* sp.	Wet	x	x	150 mL algal biomass	2.45 GHz, 900 W, 3 min	5 mg/L FAMEs	34.3	[29]
*Arthrospira platensis*	Dry	Methanol:ethyl acetate:light petroleum (1:1:1)	17:1	20 g powder (milled with mortar and pestle)	400 W, 70 °C, 1 bar, 15 min	1.59% DW FAs	x	[74]
*Chlorella vulgaris*	Dry	Chloroform:methanol (1:1)	7:1	5 g algae	700 W, 50 s (10 s on, 30 s off cycle)	31.7% DW lipid	2.39 Wh/g	[75]
*Chlorella sorokiniana*	Dry	x	10:1	1 g algae, 5 g hydrogen sulphate ionic liquid, 2% HCl	800 W, 120 °C, 60 min	27% DW lipid	x	[62]
*Nannochloropsis salina*						14% DW lipid		
*Galdieria sulphuraria*				1 g algae, 5 g hydrogen sulphate ionic liquid		22% DW lipid		
*Scenedesmus obliquus* FR751179.1	Dry	Chloroform:methanol (2:1)	20:1	1 g algae, 20 mL chloroform:methanol	1000 W, 100 °C, 10 min	16.53% DW lipid	x	[76]
*Scenedesmus obliquus*	Dry	Chloroform:methanol (1:1)	20:1	1 g algae, 20 mL chloroform:methanol	1000 W, 100 °C, 10 min	19.25% DW lipid	x	[56]
	Wet	Chloroform:ethanol (1:1)				10.08% DW lipid		
*Chlorella* sp.	Wet	x	x	15 g/L dewatered cells (15 mL distilled water), protonic ionic liquid (10:1 ratio to sample)	700 W, 3 min	3.5% DW lipid (cell disruption 74.75%)	x	[30]
*Chlorococcum* sp.						0.803% DW lipid (cell disruption 70.03%)		

x stands for no information available or provided in the research papers surveyed.

**Table 3:** Lipids extracted and directly transesterified from microalgae by MW for biodiesel production.

Microalgal Strain	Dry/Wet Method	Solvents Used	Ratio (Solvent to Sample)	Volumes Added	Microwave Settings	Product & Yield	Energy Use (MJ/kg)	Ref.	
*Nannochloropsis* sp.	Dry	Methanol:chloroform (1:2)	x	1 g of algae	2.45 GHz, 1100 W (70% power), 60 °C, 5 min (cycle mode: 21 s on, 9 s off)	32% biodiesel	x	[77]	
*Nannochloropsis salina*	Dry	Methanol	1:9	2 g algae, 24 mL methanol, 2% KOH catalyst	2.45 GHz, 800 W (50% power), 60–64 °C, 6 min	80.13% FAMEs	127	[8]	
*Nannochloropsis salina*	Dry	Methanol	1:15	3% KOH catalyst	1400 W, 1400 W, 10 min	40.03% DW FAMEs	x	[78]	
*Nannochloropsis salina*	Wet	Ethanol	9:1	2 g algae, 18 mL ethanol	1400 W reduced to 800 W, 245–285 °C, 65–80 bar, 30 min	30.9% DW FAMEs	x	[79]	
*Chlorella* sp.	Dry	Ethanol	12:1	4 g algae, 48 mL ethanol and 2% NaOH catalyst	700 W (50% power), 78 °C, 6 min	17.11% DW FAMEs	x	[70]	
Mixed microalgal culture	Dry	Methanol	8:1	5 g algae, KFCaO catalyst, 40 mL methanol	2.45 GHz, 10–80 W, 60 °C, 45 min	58.12% biodiesel	x	[80]	
*Phaeodactylum tricornutum*	Dry	Methoxide	12:1	5 g algae, 60 mL methoxide, 2% NaOH catalyst in methanol	2.45 GHz, 800 W, 1 bar, 4 min	52% biodiesel conversion efficiency	x	[81]	

x stands for no information available or provided in the research papers surveyed.

**Table 4:** Pigments extracted from microalgae by MAE.

Microalgal Strain	Dry/Wet Method	Solvents Used	Ratio (Solvent to Sample)	Volumes Added	Microwave Settings	Product & Yield	Energy Use (MJ/kg)	Ref.
*Haematococcus pluvialis*	Dry	Ethanol:ethyl alcohol (2:1)	49:1	9.81 mL solvent 200 mg algae powder	141 W, 5 min 30 s	0.59% DW astaxanthin	x	[90]
*Haematococcus pluvialis*	Dry	Acetone	100:1	0.1 g algae, 10 mL acetone	2.45 GHz, 60% of 1200 W output, 75 °C, 5 min	74% astaxanthin recovery	x	[89]
*Dunaliella tertiolecta*	Dry	Acetone	600:1	50 mg algae, 30 mL acetone	50 W, 56 °C, 1 bar, 3–5 min	0.12% DW β-carotene; 0.45% DW chlorophyll-a; 0.13% DW chlorophyll-b	x	[88]
*Cylindrotheca closterium*						0.42% DW fucoxanthin		
*Arthrospira platensis*	Dry	Methanol:ethyl acetate:light petroleum (1:1:1)	16.7:1	20 g power (milled with mortar and pestle)	400 W, 70 °C, 1 bar, 15 min	4.27% DW fatty acids	x	[74]
						0.063% DW carotenoids		
*Haematococcus pluvialis*	Dry	Acetic ether	100:1	5 g algae, 500 mL solvent, 10 mL distilled water, 10 mL n-hexane	2.45 GHz, 45 °C, 30 min	7.96 mg/100 mg astaxanthin (36.88% yield)	x	[38]
*Arthrospira platensis*	x	Ethanol:ammonium acetate (10 mM) (4:1)	x	x	400 W, 60 °C, 1 bar, 15 min	0.014% DW β-carotene	x	[91]
*Phaeodactylum tricornutum*	Dry	Ethanol	20:1	0.5 g algae, 10 mL ethanol	2.45 GHz, 850 W, 30 °C, 2 min	4.51% DW carotenoids & 0.46% DW fucoxanthin (32.26% recovery)	x	[14]

x stands for no information available or provided in the research papers surveyed.

**Table 5:** Other high-value products extracted from microalgae by MAE.

Microalgal Strain	Dry/Wet Method	Solvents Used	Ratio (Solvent to Sample)	Volumes Added	Microwave Settings	Product & Yield	Energy Use (MJ/kg)	Ref.
Unknown microalga	Wet	x	x	150 mL thickened algal biomass	900 W, 98 °C, 3 min	307.11 mLmethane/g total volatile solids	65.4	[107]
*Porphyridium purpureum*	Dry	x	x	20 mg algae, 7 mL deionised water	2.45 GHz, 40 °C, 10 s	7.37% DW phycoerythrin	x	[104]
					2.45 GHz, 100 °C, 10 s	3.48% DW phycocyanin		
					2.45 GHz, 100 °C, 1 min	3.51% DW allophycocyanin		
*Stigeoclonium* sp.; *Monoraphidium* sp.; *Nitzschia* sp. and *Navicula* sp.	Wet	x	x	150 mL algal biomass	2.45 GHz, 900 W, 3 min	915 mg/L soluble carbohydrates; 127.7 mL/g volatile solids & 193 mg/L protein	34.3	[29]
*Arthrospira platensis*	Dry	Methanol:ethyl acetate:light petroleum (1:1:1)	16.7:1	20 g power (milled with mortar and pestle)	400 W, 70 °C, 1 bar, 15 min	0.000246% DW α-tocopherol	x	[74]
*Arthrospira platensis*	Dry	Limonene:ethyl acetate (0.81:1)	7:1	Unknown starting biomass concentration	400 W, 60 °C, 1 bar, 15 min	0.085% DW thiamine	x	[91]
						0.01% DW riboflavin		
		Ethanol:ammonium acetate (10 mM) (4:1)				0.23% DW C-phycocyanin		

x stands for no information available or provided in the research papers surveyed.
